# Sickness absence among employees of the State Court of Campo Grande, Mato Grosso do Sul, Brazil

**DOI:** 10.5327/Z1679443520190473

**Published:** 2019-12-01

**Authors:** Giannine Roberta Marcelino de Souza França, Sebastião Junior Henrique Duarte, Mariluci Camargo Ferreira da Silva Candido, Adailson da Silva Moreira, Elenir Rose Jardim Cury Pontes

**Affiliations:** 1 Master’s Program in Nursing, Federal University of Mato Grosso do Sul - Campo Grande (MS), Brazil. Universidade Federal de Mato Grosso do Sul Master’s Program in Nursing Federal University of Mato Grosso do Sul Brazil

**Keywords:** occupational health, sick leave, absenteeism

## Abstract

**Background:**

The state of health of civil servants is a focus of much interest to public organizations. Within this context, health promotion, protection and recovery pose a challenge to nursing professionals engaged in occupational health surveillance.

**Objective:**

To identify the main reasons of sickness absence among employees of the State Court of Campo Grande, Mato Grosso do Sul, Brazil, in the period from 2014 through 2016.

**Methods:**

Documentary, exploratory and quantitative study in which variables of interest were sex, age, position, department, leave type, International Classification of Diseases (ICD-10) codes and duration of sick leave spells. The significance level was set to p=0.05 and the corresponding 95% confidence interval.

**Results:**

Diseases of the musculoskeletal system and connective tissue (17.8%), mental and behavioral disorders (14.5%) and factors influencing health status and contact with health services (6.2%) were the main reasons for sickness absence. The absenteeism rate along the analyzed period was higher for women (74.7%), employees aged 41 to 60 (63.5%) and judicial analysists (71.8%).

**Conclusion:**

Musculoskeletal and mental disorders were the leading causes of sickness absence among employees of the State Court of Campo Grande. The results of the present study might help health managers improve the working conditions of this population of workers.

## RESUMO |

**Introdução:**

A saúde dos servidores públicos tem sido um tema de suma importância no âmbito das empresas públicas. Sendo assim, a enfermagem, inserida no campo da vigilância em saúde do trabalhador, enfrenta o desafio da promoção, proteção e recuperação da saúde desse grupo.

**Objetivo:**

Identificar os principais motivos que levaram ao absenteísmo por doença dos servidores do Fórum da Justiça Estadual de Campo Grande (MS), entre 2014 e 2016.

**Método:**

Pesquisa de caráter documental e exploratória de análise quantitativa, utilizando as variáveis sexo, faixa etária, idade, cargo, unidade de trabalho, tipo de licença, Classificação Estatística Internacional de Doenças e Problemas Relacionados com a Saúde (CID) e número de dias de afastamento. Foram considerados nas análises p=0,05 e o intervalo de confiança de 95% (IC95%).

**Resultados:**

As doenças do sistema osteomuscular e do tecido conjuntivo (17,8%), os transtornos mentais e comportamentais (14,5%) e os fatores que influenciam o estado de saúde e o contato com os serviços de saúde (6,2%) foram as principais causas de afastamento. O absenteísmo por doença de 2014 a 2016 foi maior em mulheres (74,7%) com faixa etária de 41-60 (63,5%), bem como entre o cargo de analista judiciário (71,8%).

**Conclusões:**

O estudo demonstrou que as doenças osteomusculares e os transtornos mentais foram as principais causas de absenteísmo por doença no Fórum Judicial Estadual do município de Campo Grande (MS) e poderá contribuir no futuro para a tomada de decisões dos gestores para que possam melhorar as condições de trabalho dos servidores.

## INTRODUCTION

The world of work and organizations underwent substantial changes over time. Historically and etymologically, the word *work* is associated with unpleasantness - pain, punishment, suffering and torture, among other connotations^1^.

In 1948, together with the adoption of the Universal Declaration of Human Rights by the United Nations (UN), work became a fundamental and universal right and fair and favorable conditions should be ensured to workers. Article 25 states “Everyone has the right to a standard of living adequate for the health and well-being of himself and of his family”^2^. While this document was under preparation, discussions were held at UN to include, side by side with the civil and political also economic, social and cultural rights, which provide the necessary grounds for their effective usufruct, a condition indispensable for true human freedom^3^.

Occupational health gained visibility in Brazil following the creation of the Unified Health System, in 1988, which linked public health, risk prevention and health promotion together with the active participation of workers within a collective perspective^4^. A new Constitution promulgated that same year made occupational health a universal constitutional right^5^.

According to the European Foundation for the Improvement of Living and Working Conditions, the term absenteeism designates uninterrupted absences from work, which are computed from the day they start regardless of duration^6^. Absenteeism is the most damaging factor to the adequate operation of work processes, and thus it causes not only financial losses to organizations, but also those derived from the reorganization of the staff to cover for missing employees, the overload of work to the present ones and delays in service delivery^7^.

In Brazil, the National Justice Council implemented initiatives within a broader scoped social movement concerned with the humanization of health and labor relationships. The reason is that society is undergoing deep transformations as a function of new epidemiological paradigms and the increase in the rates of chronic degenerative diseases. As a result, health promotion poses a challenge in the case of judiciary employees^8^.

The aim of the present study was to establish the main reasons for absenteeism, rate of sickness absenteeism, total number of missed work days and mean duration of absences among employees of the State Court of Campo Grande, Minas Gerais, Brazil, from 2014 through 2016.

## METHODS

The present cross-sectional, exploratory and quantitative study involving analysis of secondary data was performed from June through October 2017 at the Heitor Medeiros State Court in Campo Grande. It was approved by the research ethics committee of Federal University of Mato Grosso do Sul in compliance with ethical principles for research involving human beings, Certificate of Presentation for Ethical Appraisal no. 58752016.3.0000.0021. All the participants signed an informed consent form.

Sample characterization data were obtained from the court’s Human Resources System and entered on an ad hoc form. We considered the following variables for analysis: sex, age, educational level, marital status, children, position, department, employment status, time in the job, type of leave, International Classification of Diseases (ICD-10) code and duration of leave spells.

We investigated associations between variables was investigated by means of the χ^2^ test and the χ^2^ test for trend and calculated prevalence ratios; with the corresponding 95% confidence interval (CI). Cox regression was used to calculate adjusted prevalence ratios (time=1 unit) for variables with significance of less than 20%. Comparison of leave spell duration between the analyzed years was performed with the non-parametric Kruskal-Wallis test (coefficient of variance >20%).

We had resource to indicators recommended by the Subcommittee on absenteeism of the International Association on Occupational Health. Absenteeism^9^ and data in the scientific literature were compared according to the number of individuals and number and duration of spells as per the following equations:

- Frequency index (workers) = number of workers granted at least one sick leave along the analyzed period/number of active workers on the last day of the year x 100^10^;
- Mean duration of leave spells = duration (in days) of spells/number of spells^11^;
- Absenteeism rate = duration of sick leave spells (in days) X 100/number of active workers in the past year X number of work days in one year (365 days)^10^.

The number of work days in one year - needed to calculate the absenteeism rate - was calculated for 365 days, because leave spells include weekends and holidays^10^.

We considered for analysis all records of workers with at least one year in the job granted sick leave for a minimum of 3 days in the period from 2014 through 2016. We excluded all other types of leave and records with missing data. Statistical analysis was performed with software Epi Into 7 (Centers for Diseases Control and Prevention, Atlanta, GA, United States) and Bioestat 5.3 (Instituto Mamirauá, Belém, Pará, Brazil).

## RESULTS

A total of 483 employees of the analyzed court were granted sick leave along the analyzed period. As Table 1 shows, the proportion of employees granted sick leave decreased from 22.7% in 2014 to 9.8% in 2016. However, we did not find substantial difference in the duration of spells, which was 40 to 50 days (Table 1).

**Table 1.** Descriptive analysis of civil servants granted sick leave and duration of spells, State Court, Mato Grosso do Sul, Campo Grande (MS), 2014-2016 (n=483).

Year	Number of employees	Employees on leave	% of employees on leave (95%CI)	Spell duration (days)	Spell duration (days) Mean±SD
2014	865	196	22.7 (19.9-25.4)	7,904	40.4±61.9
2015	862	204	23.7 (20.8-26.5)	9,876	48.4±62.6
2016	851	83	9.8 (7.8-11.7)	4,224	50.9±75.7

SD: standard deviation. Difference in mean spell duration was not significant (p=0.068, Kruskal-Wallis test); 95%CI: confidence interval of 95%.

Of 483 employees granted sick leave, 74.7% were female (95%CI 70.6-78.5%), 63.5% aged 41 to 60 (95%CI 59.1-67.8%), 30.9% had completed higher education (95%CI 36.4-45.3%) and 40.8% graduate studies (95%CI 53.0-62.0%). About 57.6% of the sample was married/in a civil union (95%CI 55.1-64.0%), 59.6% had one or two children (95%CI 67.6-75.8%), 71.8% worked as judicial analysists (55.1-64.0%), 84.1% were tenured (80.4-87.2%) and 36.4% had been 5 to 15 years in the job (32.2-40.9%) (Table 2).

**Table 2.** Number and percentage of employees granted sick leave according to variables of interest, State Court, Mato Grosso do Sul, Campo Grande (MS), 2014-2016 (n=483).

Variables	N	%	95%CI
Sex			
Female	361	74.7	(70.6-78.5)
Male	122	25.3	(21.5-29.4)
Age			
Not reported	1	0.2	(0.0-1.3)
26-40 years old	151	31.3	(27.2-35.6)
41-60 years old	307	63.5	(59.1-67.8)
Above 60 years old	24	5.0	(3.3-7.4)
Educational level			
Not reported	1	0.2	(0.0-1.3)
Incomplete elementary school	2	0.4	(0.1-1.7)
Complete elementary school	3	0.6	(0.2-2.0)
Complete secondary school	116	24.0	(20.3-28.1)
Incomplete secondary school	15	3.1	(1.8-5.2)
Complete higher education	149	30.9	(26.8-35.2)
Graduate studies	197	40.8	(36.4-45.3)
Marital status			
Married/civil union	278	57.6	(53.0-62.0)
Single	123	25.4	(21.7-29.6)
Divorced	66	13.7	(10.8-17.1)
Widowed	7	1.4	(0.6-3.1)
Other	9	1.9	(0.9-3.6)
Children			
Not reported	1	0.2	(0.0-1.3)
None	115	23.8	(20.1-27.9)
1 or 2	288	59.6	(55.1-64.0)
3 or more	79	16.4	(13.2-20.0)
Position			
Not reported	8	1.7	(0.8-3.4)
Judicial analyst	347	71.8	(67.6-75.8)
General services	41	8.5	(6.2-11.4)
High-level technician	40	8.3	(6.1-11.2)
Judicial assistant	28	5.8	(4.0-8.4)
Clerk	19	3.9	(2.5-6.2)
Employment status			
Not reported	1	0.2	(0.0-1.3)
Tenured	406	84.1	(80.4-87.2)
Retired	70	14.5	(11.5-18.0)
Active	3	0.6	(0.2-2.0)
Temporary	3	0.6	(0.2-2.0)
Years in the job			
5	43	9.0	(6.6-11.9)
>5 to 15	176	36.4	(32.2-40.9)
>15 to 25	132	27.3	(23.5-31.6)
>25	132	27.3	(23.5-31.6)

95%CI: confidence interval of 95%.

As Table 3 shows, 86 (17.8%) employees were granted sick leave for diseases of the musculoskeletal system and connective tissue, 70 (14.5%) for mental and behavioral disorders - ICD-10 codes F, 30 (6.2%) for factors influencing health status and contact with health services, 25 (5.2%) for injury, poisoning and certain other consequences of external causes and 15 (3.1%) for diseases of the digestive system. ICD-10 codes were provided for 321 employees and missed for the other 162 (33.5%). The shortest leave lasted 3 days, mean duration was 40 to 50 days.

**Table 3.** Number and percentage of employees granted sick leave according to International Classification of Diseases (ICD-10) codes, State Court, Mato Grosso do Sul, Campo Grande (MS), 2014-2016 (n=483).

ICD10	N	%	95%CI
Not reported	162	33.5	(29.4-38.0)
Diseases of the musculoskeletal system and connective tissue	86	17.8	(14.6-21.6)
Mental and behavioral disorders	70	14.5	(11.4-17.6)
Factors influencing health status and contact with health services	30	6.2	(4.3-8.9)
Injury, poisoning and certain other consequences of external causes	25	5.2	(3.5-7.7)
Diseases of the digestive system	15	3.1	(1.8-5.2)
Diseases of the eye and adnexa/Diseases of the ear and mastoid process	14	2.9	(1.7-4.9)
Diseases of the genitourinary system	13	2.7	(1.5-4.7)
Diseases of the respiratory system	11	2.3	(1.2-4.2)
Diseases of the circulatory system	10	2.1	(1.1-3.9)
Pregnancy, childbirth and the puerperium	9	1.9	(0.9-3.6)
Symptoms, signs and abnormal clinical and laboratory findings, not elsewhere classified	9	1.9	(0.9-3.6)
Certain infectious and parasitic diseases	8	1.7	(0.5-2.8)
Diseases of the nervous system	5	1.0	(0.4-2.5)
Neoplasms/Diseases of the blood and blood-forming organs and certain disorders involving the immune mechanism	4	0.9	(0.6-1.1)
Endocrine, nutritional and metabolic diseases	2	0.4	(0.1-1.7)
Diseases of the skin and subcutaneous tissue	2	0.4	(0.1-1.7)
Congenital malformations, deformations and chromosomal abnormalities	1	0.2	(0.0-1.3)

95%CI: 95% confidence interval.

A total of 196 sick leave benefits (sickness absence) were granted in 2014, corresponding to 22.7% of the employees, to a total of 7,904 days and absenteeism rate of 2.52%. A total of 204 sick leave benefits were granted in 2015, corresponding to 23.7% of the employees, to a total of 9,876 and absenteeism rate of 3.2%. A total of 83 sick leave benefits (sickness absence) were granted in 2016, corresponding to 9.8% of the employees, to a total of 4,224 days and absenteeism rate of 1.35% (Table 4).

**Table 4.** Sickness absenteeism, State Court, Mato Grosso do Sul, Campo Grande (MS), 2014-2016 (n=483).

Sickness absenteeism			
Year	2014	2015	2016
Number of spells	196	204	83
Total missed work days	7,904	9,876	4,224
Sickness absenteeism rate	2.52%	3.2%	1.35%
Mean duration (days)	40	49	50

## DISCUSSION

In the present study we analyzed official data to draw the profile of 483 employees of the State Court of Campo Grande granted sick leave in the period from 2014 through 2016. Thus we were able to identify and suggest prevention and health promotion actions for subgroups of workers more likely to miss work days due to illness.

According to the results, the total number of employees slightly decreased, from 865 to 851, along the analyzed period. Data collected by the Brazilian Institute of Geography and Statistics indicate that the population of Campo Grande increased from 786,797 in 2010 to 874,210 in 2017^12^. Therefore, since the general population grew while the number of civil servants decreased, the latter’s workload might have had increased leading to sickness absence. These findings might contribute to the planning of prevention and health promotion actions, as well as to the management of resources in a way to prioritize the most vulnerable groups^11^.

Sick leave was most often granted to women aged 41 to 60 (63.5%, 95%CI 59.1-67.8%), as also other authors reported^11^^,^^13^^,^^14^^,^^15^^,^^16^. Many women enter the workforce to improve their family income, resulting in work overload^15^. Women are more susceptible to family aspects likely to interfere with work, which fact somehow accounts for the larger prevalence of sickness absence among them by comparison to men^14^.

In a study performed at a state university the duration of sick leave spells was longer for women (73.9%)^17^. Mean duration of leaves was longer than that reported for Curitiba, Brazil, 23 days^18^. The rate of absenteeism found in the present study was smaller than that of employees of the Curitiba municipal government in the period from 2010 to 2015, 4.88% (2015). A similar finding (5.63%) was reported in a study performed at the payments department of a health care company in Brasília, Brazil^19^. A report from 2016 by the National Justice Council shows that the highest rate of sickness absence in Brazil corresponded to the State Court of Bahia, 6.3%, and the lowest to the State Court of Mato Grosso do Sul, 0.1% - versus 1.35% among the employees of the State Court of Campo Grande, in the same state.

According to Mendes and Dias^20^, the arrival of new technologies in the world of work, especially those related to automation and informatization, was not essentially meant to improve the quality of work life, but production. Indeed, such technologies pose new risks to health, often derived from the organization of work and thus difficult to approach and neutralize. The high rate of sick leave due to musculoskeletal disorders we found might be related to a large number of lawsuits, understaffing, new biotechnologies, transfer of hazardous technologies and aging of the workforce^21^^,^^22^^,^^23^^,^^24^.

The rate of sick leave spells due to musculoskeletal disorders we found differs from that reported by other authors and that in the survey performed by the National Justice Council, 13.5% - representing the second leading cause of sickness absence among judges and state judiciary employees. According to the World Health Organization^22^, work-related musculoskeletal disorders are problems involving the locomotor apparatus induced or aggravated by work and include all forms of ill-health ranging from light, transitory disorders to irreversible, disabling injuries. They are characterized as painful and harmful conditions, with negative impact for individuals and public finance.

An ICD-10 code was not indicated for 33.5% of the analyzed sick leave spells. One possible reason is the Federal Medical Council Resolution no. 1658/2002, which in article 3, section II states that among the procedures mandatory for physicians upon issuing medical certificates, inclusion of diagnosis must be explicitly authorized by the patient^25^.

## CONCLUSION

The results of the present study might be used by health managers as grounds for decision making relative to actions to improve the working conditions through prevention and intervention programs with emphasis on the health problems which most frequently lead to sickness absence.

However, for health promotion, prevention, care and vocational rehabilitation public policies to be effective, new work processes should be designed to safeguard the health of workers including their mental health. Jurisdictional public services need to find solutions to ensure adequate working conditions, minimize work-related health problems and develop interventions to improve the state of health of workers.
